# Reducing injection intensity is associated with decreased risk for invasive bacterial infection among high-frequency injection drug users

**DOI:** 10.1186/s12954-019-0312-8

**Published:** 2019-06-17

**Authors:** Salequl Islam, Damani A. Piggott, Alberto Moriggia, Jacquie Astemborski, Shruti H. Mehta, David L. Thomas, Gregory D. Kirk

**Affiliations:** 1 Department of Epidemiology, Johns Hopkins Bloomberg School of Public Health, Baltimore, USA; 2 Department of Microbiology, Jahangirnagar University, Savar, Dhaka, 1342 Bangladesh; 3 Division of Infectious Diseases, Johns Hopkins University School of Medicine, Baltimore, USA; 4 Division of Infectious and Tropical Diseases, IRCCS San Matteo Foundation, University of Pavia, Pavia, Italy

**Keywords:** Injection drug use, Bacterial infection, PWID with high frequency, PWID with reduced frequency

## Abstract

**Background:**

Bacterial infection is a major cause of morbidity and mortality for persons who inject drugs (PWID). Injection cessation may help abrogate such infections, but maintaining complete cessation is challenging. Limited data exists on the role of reduced injection intensity on invasive bacterial infection risk. We sought to evaluate decreased risk for bacterial infections following cessation and substantive reduction in the injection intensity.

**Methods:**

Participants were persons in the AIDS Linked to the Intravenous Experience (ALIVE) cohort with initial high-frequency injection drug use (> 1 daily). Pooled logistic regression with generalized estimating equations was used to estimate risk for invasive bacterial infection (pneumonia, endocarditis, or sepsis) among participants achieving complete injection cessation or reduced injection intensity relative to those with sustained high-frequency use.

**Results:**

Of 2247 study participants with 12,469 paired study visits, complete injection cessation was achieved at 13.5% and reduced injection intensity at 25.5% of study visits. Adjusting for sociodemographics and HIV status, injection cessation was associated with a 54% reduction of bacterial infection at 3 months (odds ratio [OR] 0.46, 95% CI 0.25–0.84) and a 46% reduction at 6 months (OR 0.54, 95% CI 0.36–0.81). Reduced injection intensity was associated with a 36% reduction of infection at 3 months (OR 0.64, 95% CI 0.43–0.96) and a 26% reduction at 6 months (OR 0.74, 95% CI 0.56–0.98).

**Conclusions:**

Both complete cessation and reduced injection frequency demonstrate substantial benefit in reducing invasive bacterial infection risk among PWID. With high rates of relapse into injection use, targeting sustained reductions in drug use intensity may be a key harm reduction modality for improving clinical outcomes in this population.

**Electronic supplementary material:**

The online version of this article (10.1186/s12954-019-0312-8) contains supplementary material, which is available to authorized users.

## Introduction

People who inject drugs (PWID) are associated with increased risk of local soft tissue bacterial infection, with subsequent serious risk for invasive sepsis, pneumonia, and infective endocarditis (IE) [1–3]. Injection-associated local infection and subsequent systemic progression lead to multiple morbidities, emergency room visits, and hospitalization among PWID and result in increased healthcare expenses [4–6]. Improper cleaning of the skin before injection has been associated with increased risk of skin abscess leading to severe systemic sepsis [7, 8]. Similarly, multi-fold high risk of IE and community-acquired pneumonia were reported with the increased frequency of injection drug use [9, 10]. The proportion of infections has been shown to increase with the increased frequency of subcutaneous injection [11, 12]. Thus, drug cessation may abrogate the onset of invasive bacterial infections. However, people who inject with high frequency appear less likely to achieve complete cessation [13] and, if abstinence is achieved, remain more likely to relapse [14]. Needle and syringe programs (NSPs) and opioid substitution therapy (OST) remain a key harm reduction services for PWID [15–17]. Comparative strong association was reported for OST in reducing the risk of blood-borne infection transmission [18]. However, the impact of NSP is not fully consistent because of their high heterogeneity and weaker evidence in reducing local and systemic infection transmission [18, 19]. Little data exists on whether complete cessation of injection is necessary for significant improvement in clinical outcomes, or whether clinical gains may be similarly achieved through reductions in injection intensity. There is still a shortfall of knowledge about how a decrease in the frequency of injection may influence clinical outcomes and how quickly that might occur. Moreover, limited information is known with regard to how bacterial infections might vary among PWID at different intensities of drug uses, and this is an area that needs further clarification. Recognizing complete cessation occurs infrequently, we evaluated the risk for invasive bacterial infections including bacterial pneumonia, sepsis, and endocarditis, among people who inject daily that transitioned to substantive reduction or to complete cessation in injection intensity during the subsequent 6 months.

## Materials and methods

### Study population

Subjects aged ≥ 18 years with a history of injection drug use in Baltimore, Maryland, were enrolled in the AIDS Linked to the Intravenous Experience (ALIVE) cohort described elsewhere [20, 21]. The participants ascertained their risk behavior and the history of microbial infections, which was further confirmed by abstracting their standardized medical records. Participants assessed between December 1988 and June 2012 inclusive were analyzed retrospectively.

### Study design and data measurements

At semiannual visits, ALIVE participants completed standardized questionnaires and underwent clinical examination. Detailed information obtained at each follow-up visit included socioeconomic, behavioral, and clinical parameters for the prior 6-month period. Substance use, including alcohol, tobacco, and illicit injection and non-injection drug use, was assessed by participant self-report of behaviors in the prior 6-month period. Risk behaviors were ascertained through the use of audio computer-assisted self-interview (ACASI) software. Biospecimens such as blood, sputum, and pus were collected for testing of bacterial and viral infection followed by other associated immunologic investigations.

**Fig. 1:**
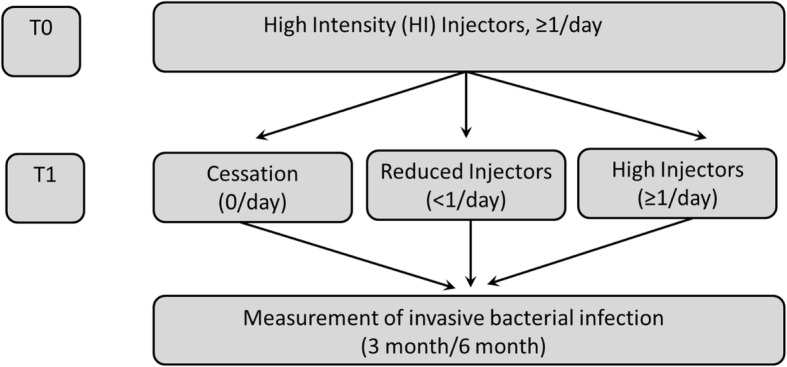
Study design for paired visit analysis. At time 0 study visit (T0), every participant was a high intensity (HI) injector (≥ 1 injection daily). Injection status over the intervening 6 months was ascertained at the next semiannual visit (T1), and participants were classified based on this latter injection status as HI injectors (continued injection use, ≥ 1 injection daily), reduced intensity (RI) injectors (< 1 injection daily), or cessation (no injection since last visit). Bacterial infection events were ascertained over the 3- and 6-month period following T1

In the dataset, all the participants were high intensity (HI) users at their index visit (time 0). At the following visit, which was approximately 6 months later and designated as T1 (time 1), their frequency of injection was categorized based on the frequency of injecting during the previous 6 months into three groups: (i) HI users (persistent heavy use), (ii) reduced intensity (RI) users (reduced use), and (iii) cessation (discontinued use). The occurrence of an invasive bacterial infection was the primary outcome assessed at 3 and 6 months subsequent to each paired visit (Fig. 1).

### Case definitions and laboratory analyses

Cases of invasive bacterial infections (sepsis, pneumonia, and IE) were identified through self-report and confirmed through standardized medical record abstraction performed by trained study nurses and approved by a physician-led outcomes committee. Biospecimens were collected at each semiannual visit. At each visit, HIV-uninfected persons had antibodies to HIV-1 assayed by enzyme-linked immunosorbent assay, with Western blot confirmation. CD4 cell counts were measured on HIV-infected persons using flow cytometry.

### Statistical analysis

We compared participant characteristics by index person visit when all the participants were in a line of high intensity (HI) users at the beginning. Pooled logistic regression with generalized estimating equations (GEE) was used to estimate the risk for invasive bacterial infections (pneumonia, sepsis, and IE) by a change in injection status for each paired visit, treating injection status as a 3-tier categorical variable as above. Sensitivity analysis was performed to evaluate possible changes of outcomes due to crossover effects of participants from one exposure group to another during the study period. Analyses were performed using SAS version 9.2 (Cary, NC).

## Results

### Participant demographics

A total of 2247 ALIVE participants contributed 12,469 person visits; 72.2% were male, 94.6% were African-American, and the remaining was mixed races of Caucasians, Native Americans, and Hispanics. About one third (32.7%) were HIV-infected at enrollment (Table 1). Additionally, 57% of participants had less than high school education, 27% were homeless, and 85% were unemployed.

**Table 1:** Participant characteristics by study visits at two timepoints and by injection status^a^

Characteristics	Study visits			
Demographic	Time 0 No. of visits, %	Time 1 No. of visits, %		
	High intensity injector, (*n* = 12,469) 100	Cessation, (*n* = 1680) 13.47	Reduced intensity, (*n* = 3185) 25.54	High intensity, (*n* = 7604) 60.98
Gender				
Male	(9006) 72.23	13.30	26.49	60.20
Female	(3463) 27.77	13.92	23.07	63.01
Race				
African-American	(11,797) 94.63	13.60	32.44	53.96
White/Hispanic	(669) 5.37	13.47	25.16	61.37
Education				
High school or more	(4957) 39.85	13.70	26.81	59.49
Less than high school	(7481) 60.15	13.31	24.66	62.02
Marital status				
Married	(4077) 32.75	13.56	26.44	60.00
Never married	(8373) 67.25	13.42	25.08	61.50
Homeless^b^				
No	(9392) 79.22	13.13	25.55	61.32
Yes	(2463) 20.78	15.87	25.90	58.22
Employment^b^				
No	(8846) 83.89	14.24	24.75	61.01
Yes	(1699) 16.11	14.42	25.13	60.45
Behavioral				
Any non-IV drug use^b^				
No	(5991) 48.16	14.54	23.80	61.66
Yes	(6448) 51.84	12.55	27.16	60.30
Types of drug^b^				
Heroin	(11,631) 93.66	11.59	23.55	58.53
Cocaine	(11,044) 88.94	11.93	25.94	62.13
Marijuana	(3885) 31.17	10.14	27.54	62.32
Cigarette smoking^b^				
No	(900) 7.24	19.78	29.22	51.00
< 2 packs/day	(10,153) 81.63	13.27	25.89	60.84
≥ 2 packs/day	(1385) 11.14	10.69	20.43	68.88
Alcohol use^b^				
No	(3246) 26.15	19.75	23.17	57.09
Drank > 1 day/week	(6490) 52.29	11.54	28.12	60.34
Drank 7 days/week	(2675) 21.55	10.65	22.21	67.14
Clinical				
HIV status				
Negative	(8265) 67.31	12.96	24.68	62.36
Positive	(4014) 32.69	14.49	27.24	58.28
CD4 counts (per mm^3^)^c^				
≥ 500	(1306) 32.54	14.32	25.57	60.11
200–499	(1857) 46.26	14.11	27.84	58.05
≤ 200	(851) 21.20	15.75	28.44	55.82

^a^Data are number (%) of study visits

^b^Characteristics in previous 6 months

^c^reflects characteristics among HIV-positive individuals only

### Drug use behaviors

Of all participants, 94% reported active heroin use and 89% active cocaine use. Non-injection drug use was observed among 52% of participants. Further, 74% reported moderate to heavy alcohol consumption and 93% tobacco use (Table 1). Of 12,469 initial high intensity injection visits, 61% had continued high intensity at the subsequent paired visit, 25.5% reported substantial decline in injection intensity, and 13.5 % complete cessation.

### Bacterial infection events

In the 3-month follow-up period, there were 161 (1.29% of paired study visits) cases of bacterial infection—105 episodes of bacterial pneumonia, 30 episodes of sepsis, and 26 episodes of infective endocarditis (Additional file 1: Table S1). Of these 161 cases, 112 (1.47%) were among HI injectors, 34 (1.07%) among RI injectors, and the remaining 15 (0.89%) among cessation participants. In the cumulative 6-month follow-up period, there were 324 (2.6%) cases of bacterial infection, including 210 episodes of bacterial pneumonia, 66 episodes of sepsis, and 52 episodes of infective endocarditis. Of these 324 cases, 218 (2.87%) were among continued HI injectors, 74 (2.32%) among reduced injection users, and 32 (1.90%) in cessation subjects.

### Reduced injection intensity lowers risk for bacterial infection

Increasing age and female gender were both significantly associated with a higher risk of bacterial infection at 3 and 6 months (Table 2). HIV infection was also significantly associated with increased odds of bacterial infection; risk notably increased with increasing degree of immunosuppression. HIV-infected participants with a CD4 count > 500 cells/ml had a 3.7-fold increased odds and 3.5-fold increased odds (95% confidence interval [CI], 2.30–5.39) of bacterial infection at 3 months and 6 months, respectively. HIV-infected participants with a CD4 count < 200 demonstrated an 11.6-fold increased odds and 10.4-fold increased odds of bacterial infection at 3 months and 6 months, respectively (Table 2).

**Table 2:** Odds of invasive bacterial infection in ALIVE participants with cessation and reduced intensity injection

Variables	3-month observation^a^				6-month observation^a^			
	Unadjusted		Adjusted^b^		Unadjusted		Adjusted^b^	
	OR (95% CI)	*p*	OR (95% CI)	*p*	OR (95% CI)	*p*	OR (95% CI)	*p*
Age (per 10 year)	1.18 (0.95–1.46)	0.135	1.30 (1.03–1.66)	0.029	1.21 (1.04–1.42)	0.012	1.34 (1.13–1.59)	0.001
Gender (female)	1.26 (0.86–1.84)	0.229	1.74 (1.17–2.57)	0.043	1.50 (1.14–1.97)	0.003	1.76 (1.34–2.32)	< 0.001
HIV status by CD4 count								
HIV negative > 500 cells/mm^3^	1.0 (Ref) 3.13 (1.72–5.70)	<0.001	1.0 (Ref) 3.71 (2.03–6.79)	< 0.001	1.0 (Ref) 3.01 (1.97-4.59)	< 0.001	1.0 (Ref) 3.52 (2.3–5.39)	< 0.001
200–499 cells/mm^3^	5.13 (3.21–8.19)	< 0.001	5.81 (3.63–9.29)	< 0.001	4.96 (3.57–6.88)	< 0.001	5.52 (3.97–7.69)	< 0.001
< 200 cells/mm^3^	11.06 (6.98–17.55)	< 0.001	11.55 (7.23–18.43)	< 0.001	10.31 (7.41–14.3)	< 0.001	10.39 (7.44–14.5)	< 0.001
Injection intensity								
High intensity	1.0 (Ref)		1.0 (Ref)		1.0 (Ref)		1.0 (Ref)	
Reduced intensity	0.74 (0.50–1.09)	0.126	0.64 (0.43–0.96)	0.031	0.81 (0.62–1.06)	0.121	0.74 (0.56–0.98)	0.035
Cessation of injection	0.62 (0.36–1.07)	0.084	0.46 (0.25–0.84)	0.012	0.65 (0.45–0.95)	0.025	0.54 (0.36–0.81)	0.003

*CI* confidence interval, *Ref* reference

^a^Data are odds ratios (95% confidence intervals)

^b^Each model adjusted for race, non-injection drug use, alcohol use, and tobacco use

**Fig. 2:**
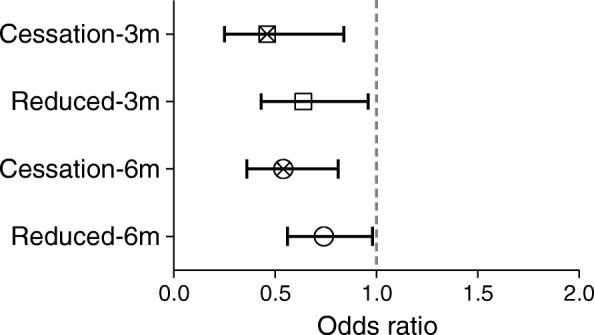
Risk of bacterial infection with cessation and with reduced intensity injection at 3 and 6 months. Data represent odds ratios and 95% confidence intervals for invasive bacterial infection risk at 3 and 6 months by change in injection intensity status. High intensity injectors (those with continued ≥ daily injection) are the reference group. Models are adjusted for age, gender, alcohol use, tobacco use, non-injection drug use, and HIV status

In multivariate analysis adjusting for demographics, HIV status, non-injection drug use, alcohol use, and smoking (Fig. 2), reduced injection intensity was significantly associated with a 36% reduction in bacterial infection at 3 months (odds ratio [OR] 0.64, 95% CI 0.43–0.96) and a 26% reduction at 6 months (OR 0.74, 95% CI 0.56–0.98) (Table 2). Cessation of injection was significantly associated with a 54% reduction of bacterial infection at 3 months (OR 0.46, 95% CI 0.25–0.84) and a 46% reduction in the cumulative 6-month period (OR 0.54, 95% CI 0.36–0.81). Again, we conducted three separate analyses to assess the breakdown effects of bacterial infections from a single outcome to pneumonia, sepsis, and infective endocarditis. Independent analysis of each infection types appeared protective ( OR 0.74 for bacterial pneumonia; OR 0.84 for sepsis, and OR 0.60 for IE) among reduced intensity PWID. Nonetheless, all the discrete analyses lost statistical power which could be for smaller individual cases. And as expected, cessation of drug uses exhibited better protection invariably (Additional file 2: Table S2). Part of our data included a harm reduction service, needle and syringe program (NSP). We conducted an independent multivariate analysis of the partial data, where NSP was found marginally protective in overall bacterial infections without statistical significance (OR 0.89 (95% CI, 0.59–1.33)). Since NSP was missing in major parts of our data, we excluded it from our primary analyses. Further, we ran a different analysis using years from the first injection instead of age in the model. While the estimate for years of use was significant, it was slightly less than that observed with age. The protective effect of reduction in drug use was essentially unchanged (Additional file 3: Table S3).

### Sensitivity analysis

We initially measured our outcome variable based on an intention-to-treat (ITT) concept by ignoring crossover non-compliance following the completion of our 6-month study period. In separate multivariate analyses based on an as-treated (AT) concept, we measured the outcome of bacterial infection from participants whose injection status remained unchanged for the 6-month period following timepoint T1. Cessation of injection was still found to be consistently protective with this approach, with an 83% reduction in bacterial infections in the following 3 months (OR 0.17, 95% CI 0.05–0.55, *p* 0.003) and a 60% reduction in the cumulative 6-month period (OR 0.40, 95% CI 0.22–0.72, *p* 0.002). In contrast, whereas there was still a trend towards protection with reduced intensity with this approach, these findings did not meet statistical significance, with OR 0.66 (95% CI, 0.36–1.22) at 3 months and OR 0.91 (95% CI 0.62–1.35) at 6 months. The attenuated effect of RI injection by AT analysis compared to ITT is probably due primarily to a reduced sample size.

## Discussion

In this study, we sought to examine the role of reduced drug injection intensity on bacterial infection risk. Our findings demonstrate significant reductions in bacterial infections among reduced intensity injectors and those who achieve complete drug cessation. Cessation of injection drug use has been the primary goal of PWID-targeted interventions to ameliorate significant drug-related morbidity with its severe medical and economic consequences. Indeed, we observed the strongest clinical benefit among PWID that achieved complete cessation. However, maintaining cessation has been highly challenging to achieve, particularly among chronic people who inject drugs with high frequency, with drug dependency and withdrawal effects often precipitating obstacles against sustained abstinence [22]. In prior studies in our cohort, we found low rates of sustained drug cessation, with up to three quarters of those initially achieving cessation reinitiating injection drug use within the following year [13, 23]. Frequent relapse after initial cessation has been similarly noted in other settings [14]. Understanding the current real-world situation, the clinical benefits associated with reduced injection intensity are observed in this study. It suggests that targeting reduction in intensity, as opposed to a singular focus on complete drug cessation, may find value as an interim goal for clinical harm reduction among chronic high intensity users.

Congruent with prior studies, we also found increasing age, HIV infection, and female gender to be independently associated with increased risk of bacterial infection [24–26]. Older adults have been known to have increased vulnerability to infection, likely mediated in part by increasing age-associated dysregulation of immune pathways [27]. Similarly, the immune dysregulation associated with HIV infection is well known and the dose-dependent relationship of increasing bacterial infection risk with more advanced immunosuppression as observed in this study has been previously well characterized [28] and provides strong face validity for the primary outcome variable in our study.

Harm reduction followed by the ultimate cessation of injection drug use has been a key public health target to improve the social and clinical well-being of the PWID population. Many studies have suggested the value of needle and syringe programs (NSPs) as part of harm reduction interventions to reduce infection among PWID [29]. However, continued infection with high intensity injection can still occur even with sterile injection equipment, as a consequence of contaminated drugs or injection of skin flora, particularly by *Streptococcal* or *Staphylococcus* species that can lead to life-threatening invasive infection [11, 30, 31]. Our findings suggest the potential role for complementary harm reduction approaches targeting both improved safety and reduced quantity of injection use.

This study does have several important limitations. Risk behavior data was collected by self-report. However, use of the ACASI system for such questions reduces the potential bias associated with participant response. Further, self-reported data from ALIVE cohort participants has been well validated in multiple prior studies [20, 32] and found to be reliable. Although ALIVE performs a comprehensive assessment of risk behavior, some relevant exposure data was not captured (e.g., groin injection site). Several outcome variables were also initially collected by self-report but confirmed by medical record review and by a physician-led endpoints committee. This study was also performed in a cohort of predominantly African-American PWID in Baltimore with a high degree of socioeconomic challenge. Further studies may be required to assess the generalizability of our findings to other PWID populations. However, our cohort represents those with the highest degree of vulnerability to morbidity and mortality who would most benefit from interventions to reduce invasive bacterial infections and improve overall clinical outcomes. Another concerned issue that the study has analyzed is the pulled data of 24 years, from 1988 to 2012. The time, 24 years, could carry some confounders like the healthcare landscape for PWID, expansion of antiretrovirals, and community-level HIV testing initiatives. The different facilities and treatment regimens for PWID might affect the infection outcomes of interest during the long period. To assess potential changes of bacterial infections over time, we conducted comparable analyses with four separate smaller sub-samples. Each sub-sample analysis showed the protective estimation of bacterial infections among reduced intensity PWID (OR 0.60 to 0.89) that has warranted our combined analysis. However, three of those four analyses lost statistical significance, which could be attributable to the reduction in sample size. A separate analysis restricted to the most recent 5-year data appeared more protective (OR 0.18), but the association was not statistically significant.

## Conclusions

In conclusion, our findings suggest high intensity injection drug use behavior is a predisposing factor for bacterial infections. Reducing injection frequency had rapid and substantial benefit in decreasing risk for serious bacterial infections even in the absence of complete cessation. Relapse frequently occurs after initial complete drug cessation due to drug dependence characteristics and remains a primary obstacle in treating drug abuse. Maintaining reduced drug injection intensity for an adequate length of time prior to cessation may find significant benefit in improving clinical outcomes and quality of life for PWID.

## Additional files

Additional file 1: **Table S1.** Episodes of bacterial infection in the 3- and 6-month period following injection status change in ALIVE. (DOCX 37 kb)

Additional file 2: **Table S2.** Multivariable relative bacterial infections among people who inject drugs (PWIDs) stratified by different intensities of drug uses and types of infections. (DOCX 12 kb)

Additional file 3: **Table S3.** Odds of invasive bacterial infection with years of drug use, cessation, and reduced intensity injection. (DOCX 15 kb)

## Data Availability

A subset dataset was generated or analyzed retrospectively from the ALIVE cohort during the current study. All the dataset has been preserved at the databank repository of the ALIVE study. Data can be shared upon request following universal data-sharing rules and as per by-law of Johns Hopkins University.

## Abbreviations

ACASI: Audio computer-assisted self-interview
AIDS: Acquired immune deficiency syndrome
ALIVE: AIDS Linked to the Intravenous Experience
AT: As-treated
CD4: Cluster of differentiation 4
CI: Confidence interval
GEE: Generalized estimating equations
HBV: Hepatitis B virus
HCV: Hepatitis C virus
HI: High intensity (injectors)
HIV: Human immunodeficiency virus
IE: Infective endocarditis
ITT: Intention-to-treat
NC: North Carolina
NIDA: National Institute of Drug Abuse
NSP: Needle and syringe program
OR: Odds ratio
OST: Opioid substitution therapy
PWID: Persons who inject drugs
RI: Reduced intensity (injectors)
T0: Initial visit time
T1: Follow-up visit time
