# Supplementary material for: Reducing injection intensity is associated with decreased risk for invasive bacterial infection among high-frequency injection drug users
Source: Harm Reduct J. 2019 Jun 17;16:38. doi: 10.1186/s12954-019-0312-8 (PMC6580632; doi:10.1186/s12954-019-0312-8)
Supplement: Supplementary file 1 — Table S1. Episodes of bacterial infection in the 3- and 6-month period following injection status change in ALIVE. (DOCX 37 kb) [file 12954_2019_312_MOESM1_ESM.docx]

**Supplementary Table 1. Episodes of Bacterial Infection in the 3 and 6 Month Period Following Injection Status Change in ALIVE**

| **Types of infection** | **Episodes of bacterial infection, No. (%)^a^** | | | | | | |
| --- | --- | --- | --- | --- | --- | --- | --- |
|  | **3 months*** | | |  | **6 months*** | | |
|  | **HI** | **RI** | **Cessation** |  | **HI** | **RI** | **Cessation** |
| Bacterial pneumonia | 73 (1.0) | 20 (0.6) | 12 (0.7) |  | 140 (1.8) | 47 (1.5) | 23 (1.4) |
| Sepsis | 21 (0.3) | 9 (0.3) | 0 (0) |  | 44 (0.6) | 18 (0.6) | 4 (0.2) |
| Infectious Endocarditis | 19 (0.3) | 5 (0.2) | 2 (0.1) |  | 38 (0.5) | 10 (0.3) | 4 (0.2) |
| Total | 112 (1.5) | 34 (1.1) | 15 (0.9) |  | 218 (2.9) | 74 (2.3) | 32 (1.9) |

^a^ Data are number (%) of 12,469 paired study visits
