# Supplementary material for: Reducing injection intensity is associated with decreased risk for invasive bacterial infection among high-frequency injection drug users
Source: Harm Reduct J. 2019 Jun 17;16:38. doi: 10.1186/s12954-019-0312-8 (PMC6580632; doi:10.1186/s12954-019-0312-8)
Supplement: Supplementary file 2 — Table S2. Multivariable relative bacterial infections among people who inject drugs (PWIDs) stratified by different intensities of drug uses and types of infections. (DOCX 12 kb) [file 12954_2019_312_MOESM2_ESM.docx]

**Supplementary Table 2**. Multivariable Relative Bacterial Infections among People Who Inject Drugs (PWIDs) Stratified by

Different Intensities of Drug-uses and Types of Infections.

| **Intensities of Drug-uses** | **Multivariate OR (95% CI)** | | | |
| --- | --- | --- | --- | --- |
|  | **Bacterial Pneumonia** | **Sepsis** | **Infectious Endocarditis** | **Combined Infections** |
| **HI users** | 1.0 [Reference] | 1.0 [Reference] | 1.0 [Reference] | 1.0 [Reference] |
| **RI users** | 0.74 (0.52, 1.04) | 0.84 (0.48, 1.48) | 0.60 (0.30, 1.23) | 0.74 (0.56, 0.97)* |
| **Cessation** | 0.65 (0.40, 1.05) | 0.32 (0.11, 0.90)* | 0.33 (0.11, 0.97) * | 0.54 (0.36, 0.81)* |

Abbreviations: CI, confidence interval; HI, persistent high intensity; RI, reduced intensity; OR, odds ratio,

*, statistically significant reduction of infections in RI users and cessation groups with p<0.05.

**Supplementary Table 2**. Multivariable Relative Bacterial Infections among People Who Inject Drugs (PWIDs) Stratified by

Different Intensities of Drug-uses and Types of Infections.

| **Intensities of Drug-uses** | **No. of observations** | **No. of events** | **Multivariate OR (95% CI)** | | | |
| --- | --- | --- | --- | --- | --- | --- |
|  |  |  | **Bacterial Pneumonia** | **Sepsis** | **Infectious Endocarditis** | **Combined Infections** |
| **HI users** | 12,212 |  | 1.0 [Reference] | 1.0 [Reference] | 1.0 [Reference] | 1.0 [Reference] |
| **RI users** |  |  | 0.74 (0.52, 1.04) | 0.84 (0.48, 1.48) | 0.60 (0.30, 1.23) | 0.74 (0.56, 0.97)* |
| **Cessation** |  |  | 0.65 (0.40, 1.05) | 0.32 (0.11, 0.90)* | 0.33 (0.11, 0.97) * | 0.54 (0.36, 0.81)* |
|  |  |  |  |  |  |  |

Abbreviations: CI, confidence interval; HI, persistent high intensity; RI, reduced intensity; OR, odds ratio,

*, statistically significant difference between HI and RI users with p<0.05.
