# Supplementary material for: Reducing injection intensity is associated with decreased risk for invasive bacterial infection among high-frequency injection drug users
Source: Harm Reduct J. 2019 Jun 17;16:38. doi: 10.1186/s12954-019-0312-8 (PMC6580632; doi:10.1186/s12954-019-0312-8)
Supplement: Supplementary file 3 — Table S3. Odds of invasive bacterial infection with years of drug use, cessation, and reduced intensity injection. (DOCX 15 kb) [file 12954_2019_312_MOESM3_ESM.docx]

**Supplementary Table 3.** Odds of Invasive Bacterial Infection with years of drug use, cessation and reduced intensity injection.

| Variables | **3-month observation ^a^** | | **6-month observation ^a^** | |
| --- | --- | --- | --- | --- |
|  | **OR (95% CI) ^b^** | **P Value** | **OR (95% CI) ^b^** | **P Value** |
| Years of injection use | 1.03 (1.01-1.06) | 0.001 | 1.03 (1.01-1.04) | 0.001 |
| High Injection intensity | 1.0 (Ref) |  | 1.0 (Ref) |  |
| Reduced Injection intensity | 0.65 (0.43-0.97) | 0.034 | 0.74 (0.56-0.98) | 0.035 |
| Cessation of injection | 0.46 (0.25-0.84) | 0.012 | 0.54 (0.36-0.82) | 0.004 |

CI, confidence interval; Ref, Reference, OR, Odds ratio

^a^ Each model adjusted for race, non-injection drug use, alcohol use and tobacco use.

**^b^** Data are odds ratios (95% confidence intervals)
